# Pt-Chitosan-TiO_2_ for Efficient Photocatalytic Hydrogen Evolution via Ligand-to-Metal Charge Transfer Mechanism under Visible Light

**DOI:** 10.3390/molecules27154673

**Published:** 2022-07-22

**Authors:** Yanru Liu, Jingyun Mao, Yiwei Huang, Qingrong Qian, Yongjin Luo, Hun Xue, Songwei Yang

**Affiliations:** 1 College of Life Sciences, Fujian Normal University, Fuzhou 350117, China; yrliu@fjnu.edu.cn (Y.L.); hywadgjmptw@163.com (Y.H.); 2 College of Environmental Science and Engineering, Fujian Normal University, Fujian Key Laboratory of Pollution Control & Resource Reuse, Fuzhou 350007, China; mjy19960427@163.com (J.M.); qrqian@fjnu.edu.cn (Q.Q.); yongjinluo@fjnu.edu.cn (Y.L.)

**Keywords:** photocatalyst, TiO_2_, chitosan, H_2_ evolution, LMCT, visible light

## Abstract

The Pt-chitosan-TiO_2_ charge transfer (CT) complex was synthesized via the sol-gel and impregnation method. The synthesized photocatalysts were thoroughly characterized, and their photocatalytic activity were evaluated toward H_2_ production through water reduction under visible-light irradiation. The effect of the preparation conditions of the photocatalysts (the degree of deacetylation of chitosan, addition amount of chitosan, and calcination temperature) on the photocatalytic activity was discussed. The optimal Pt-10%DD75-T200 showed a H_2_ generation rate of 280.4 μmol within 3 h. The remarkable visible-light photocatalytic activity of Pt-chitosan-TiO_2_ was due to the CT complex formation between chitosan and TiO_2_, which extended the visible-light absorption and induced the ligand-to-metal charge transfer (LMCT). The photocatalytic mechanism of Pt-chitosan-TiO_2_ was also investigated. This paper outlines a new and facile pathway for designing novel visible-light-driven photocatalysts that are based on TiO_2_ modified by polysaccharide biomass wastes that are widely found in nature.

## 1. Introduction

In recent years, semiconductor photocatalysis driven by solar light has had broad application prospects in the field of environmental chemistry [1,2,3,4]. As a photocatalyst for environmental purification and solar energy conversion, titanium dioxide (TiO_2_) is known for its high oxidation power, low price, chemical stability, and non-toxicity [5,6,7]. However, pure TiO_2_, with a band gap of 3.2 eV, can only be excited by ultraviolet light (<5% of solar energy), which limits its practical applications [8,9]. Therefore, many studies have been conducted to obtain visible-light-responsive TiO_2_-based photocatalysts, including dye sensitization [10,11], impurity doping [12,13,14], coupling with other semiconductors [15,16,17], etc.

Another method of visible-light activation of TiO_2_ is the formation of charge transfer (CT) complexes between TiO_2_ and organic molecules that do not absorb visible light by themselves [18]. The CT complexes generate visible-light-driven ligand-to-metal charge transfer (LMCT) that represents the transfer of the electrons from the highest occupied molecular orbital (HOMO) of adsorbates to the conduction band (CB) of TiO_2_, which changes its optoelectronic properties [19]. Up to now, organic molecules such as ethylene diamine tetraacetic acid (EDTA) [20], phenol [21], catechol [22], dopamine [23], and glucose [24] have been reported as surface ligands for the modification of TiO_2_ to extend its spectrum response range into the visible-light region. For instance, EDTA-TiO_2_, a typical LMCT complex, induces a visible-light absorption of 550 nm and can be applied to reduce Cr(VI) to Cr(III) under visible-light irradiation, which can be attributed to the LMCT mechanism, where the visible-light irradiation directly excites electrons from the HOMO of EDTA to the conduction band (CB) of TiO_2_ [25]. Kim et al. reported that glucose absorbed on the surface of TiO_2_ nanoparticles can easily form complexes with TiO_2_ via their hydroxyl groups. Glucose-TiO_2_ complexes showed photocatalytic activities for the reduction of Cr(VI) to Cr(III) and O_2_ to H_2_O_2_ under visible-light irradiation, although neither glucose nor TiO_2_ can absorb visible light [26]. Li et al. demonstrated that the hydroxyl groups on a cellulose nanocrystal (CNC) can guide the growth of TiO_2_ nanoparticles on it and induce the formation of CT complexes. TiO_2_/CNC nanocomposites show an excellent performance for photoreduction of Cr(VI) under visible light [27]. Chitosan, derived from the partial deacetylation of chitin that is extracted from the shell of marine crustaceans, possesses functional groups such as the amino group and hydroxyl group [28]. The degree of deacetylation of chitosan signifies the proportion of free amino groups in chitosan. The higher the degree of deacetylation and the larger the quantity of acetyl groups removed from acetyl amino groups, the more obvious the chemical properties of the amino groups. Chitosan, being rich in natural resources and low in cost, has been widely applied in the photocatalytic field and absorbing heavy metal ions [29,30]. Li et al. fabricated chitosan/g-C_3_N_4_/TiO_2_ nanofibers for Cr(VI) removal through adsorption and photocatalytic processes [30]. Balakrishnan et al. immobilized TiO_2_ on chitosan to achieve the recyclable photocatalyst that exhibits an excellent 2,4-dichlorophenoxyacetic acid performance under UV light [31]. However, chitosan and TiO_2_ complex for H_2_ evolution under visible-light irradiation by a LMCT mechanism has not been reported.

In this study, Pt-chitosan-TiO_2_ LMCT complexes were prepared and employed as photocatalysts for H_2_ production under visible-light irradiation. Synthetic conditions were optimized to achieve the highest photocatalytic activity, including the optimization of different degrees of deacetylation of chitosan, temperature of preparation, and the amount of chitosan. The as-obtained Pt-chitosan-TiO_2_ LMCT complexes achieved a high H_2_ evolution rate of 280.4 μmol within 3 h under visible-light irradiation. Furthermore, we present the photocatalytic mechanism of Pt-chitosan-TiO_2_ LMCT complexes.

## 2. Results and Discussion

### 2.1. Physicochemical Properties of the Obtained Samples

**Figure 1:**
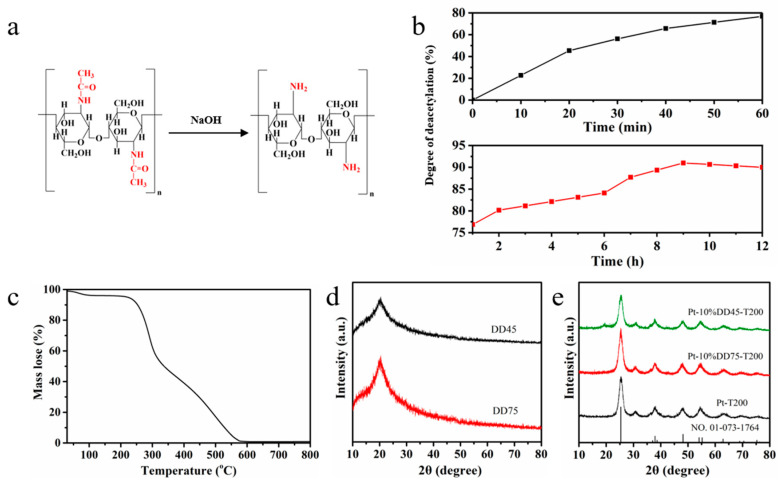
(**a**) Graph of degree of deacetylation of chitosan varying with the reaction time; (**b**) reaction of the deacetylation of chitin; (**c**) TGA curve of chitosan; (**d**) XRD patterns of DD45 and DD75, and (**e**) XRD patterns of Pt-T200, Pt-10%DD45-T200, and Pt-10%DD75-T200.

The reaction process of the deacetylation of chitin is shown in Figure 1a. Chitosan was obtained by removing some or all of the acetyl groups from chitin after high-temperature treatment with concentrated alkali. Figure 1b shows that the deacetylation degree of chitosan varied with the reaction time. The change in deacetylation degree presented a rapidly increasing trend within 60 min. The trend slowly increased after 60 min, which we attributed to the increasing number of acetyl groups in the solution during the reaction process. Figure 1c presents the TGA curve of the chitosan. There are two stages on the curve of the chitosan from 30 to 800 °C. A weight loss of about 3.8% between 30 and 100 °C was due to the loss of water molecules in chitosan. The enormous weight loss of 94.2% between 230 and 570 °C was ascribed to the decomposition of the organic component in chitosan [32]. The results indicated that the chitosan can remain unchanged when the temperature is below 230 °C.

The XRD patterns of DD45 and DD75 are depicted in Figure 1d. The broad peaks at 20.5° were attributed to (110) the plane of chitosan [30]. The intensity of the diffraction peaks decreased with the increase in the degree of deacetylation, which is in accordance with the literature [33]. The XRD patterns of Pt-10%DD75-T200, Pt-10%DD45-T200, and Pt-T200 are shown in Figure 1e. All of the samples exhibited diffraction peaks corresponding to standard anatase TiO_2_ (JCPDS No.01-073-1764) [34]. A diffraction peak at 19.9° was indexed to chitosan in Pt-10%DD45-T200. However, the peak attributed to chitosan disappeared in the XRD pattern of Pt-10%DD75-T200, which was due to the complete dissolution of DD75 in TiO_2_ sol. Compared with DD45, DD75 had better solubility in TiO_2_ sol, which is more conducive for forming CT complexes with TiO_2_. Furthermore, the average crystal diameters of Pt-T200, Pt-10%DD45-T200, and Pt-10%DD75-T200 were calculated from the Scherrer equation, based on the half-width of the strongest diffraction peak of anatase TiO_2_. The results are 6.00, 5.45, and 5.42 nm, which demonstrated that the degrees of deacetylation have little effect on the microstructure of TiO_2._

**Figure 2:**
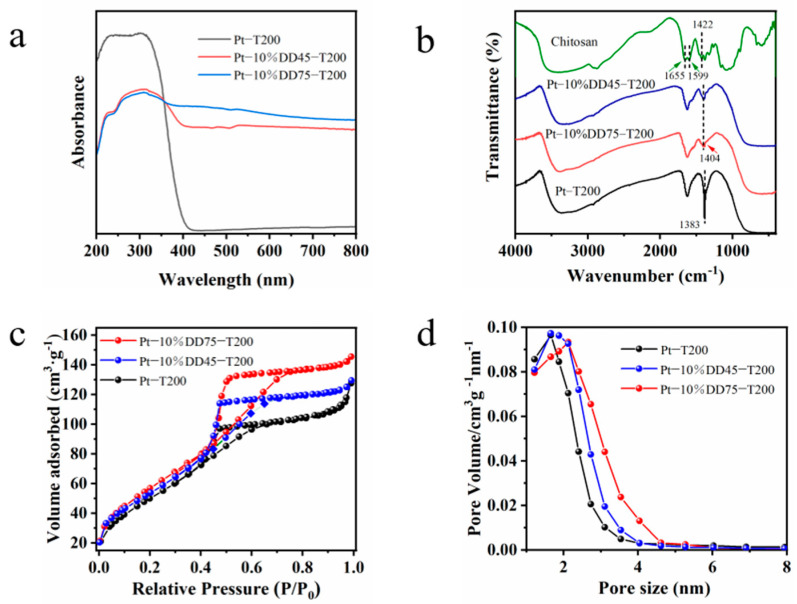
(**a**) UV–vis diffuse reflectance spectra, (**b**) FT-IR spectra, (**c**) N_2_ adsorption-desorption isotherms, and (**d**) BJH pore size distribution of as-prepare Pt-Chitosan-TiO_2_ samples.

The UV–vis diffuse reflectance spectra of DD45, DD75, Pt-T200, and the as-prepared Pt-chitosan-TiO_2_ samples are displayed in Figure S1a–c and Figure 2a. As can be seen, the Pt-T200, DD45, and DD75 samples showed no visible-light absorption, and Pt-T200 only showed an absorption edge at about 393 nm, indicating that it can only be excited by UV light. On the other hand, Pt-chitosan-TiO_2_ CT complexes extended the absorption from the UV region to the visible-light region. In addition, the absorption edges of Pt-DD45-T200 and Pt-DD75-T200 were 558 and 778 nm, respectively. Such a shift was attributed to the formation of the LMCT complex between chitosan and TiO_2_.

The enhanced visible absorption was attributed to the formation of the CT complex, which may increase the photocatalytic activity under visible-light irradiation [27].

The FT-IR spectra of the chitosan, Pt-10%DD45-T200, Pt-10%DD75-T200, and Pt-T200 are shown in Figure 2b. In the FT-IR spectrum of Pt-T200, the stretching and bending of O–H near 3370 cm^−1^ was observed [8]. The peak at 1383 cm^−1^ was assigned to the –CH_2_ and –CH_3_ (due to the incomplete hydrolysis of tetrabutyl titanate), and the peaks from 400 to 800 cm^−1^ related to the typical vibration of the O–Ti–O bonding of the TiO_2_ structure [34]. With respect to chitosan, the peak at 3415 cm^−1^ was attributed to the stretching vibration of both O–H and –NH_2_; the peaks located around 1655 and 1599 cm^−1^ were attributed to the amide I group and –NH deformation, respectively. The peak at 1422 cm^−1^ was due to the bending vibration of –NH_2_ [35]. As for Pt-10%DD45-T200 and Pt-10%DD75-T200, the peaks located at 1404 cm^−1^ were assigned to the –CH_2_, –CH_3_, and the bending vibration of –NH_2_. All the main characteristic peaks of TiO_2_ and the bending vibration of –NH_2_ appeared in the FT-IR spectra of Pt-10%DD75-T200 and Pt-10%DD45-T200, indicating that the chitosan and TiO_2_ composites were successfully synthesized.

The N_2_ adsorption-desorption isotherms (Figure 2c) show that all three samples (Pt-10%DD75-T200, Pt-10%DD45-T200, and Pt-T200) displayed type-IV isotherms, which is a typical adsorption behavior of mesoporous materials. Furthermore, Pt-10%DD75-T200 (221.9 m^2^/g^1^) showed a higher specific surface area than that of Pt-10%DD45-T200 (208.6 m^2^/g^1^) and Pt-T200 (198.4 m^2^/g^1^). The average pore sizes of Pt-10%DD75-T200, Pt-10%DD45-T200, and Pt-T200 are about 4.1, 3.8, and 4.0 nm (Figure 2d), respectively. This increased specific surface area can increase the contact area between reactants and photocatalysts, leading to higher photocatalytic activity.

**Figure 3:**
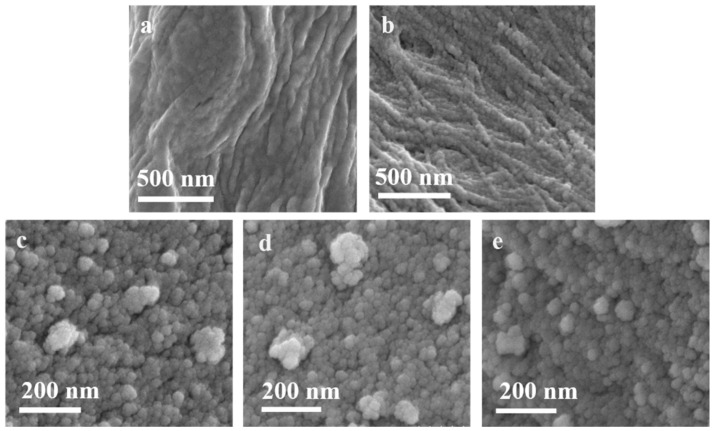
SEM images of (**a**) DD45, (**b**) DD75, (**c**) Pt-T200, (**d**) Pt-10%DD45-T200, and (**e**) Pt-10%DD75-T200.

The SEM images of DD75 and DD45 are shown in Figure 3a,b. Both of them were composed of particles with homogeneous, compact, and wrinkled surfaces. In addition, the interface between DD45 particles was more distinct than that between DD75 particles. As shown in Figure 3c, the SEM image of Pt-T200 depicts that it entirely consisted of nanoparticles. As shown in Figure 3d,e, the SEM images of Pt-DD75-T200 and Pt-DD45-T200 both reveal a uniform nanoparticle morphology similar to that of TiO_2_. However, the aggregation phenomenon of Pt-DD75-T200 nanoparticles was less than that of Pt-T200 and Pt-DD45-T200, suggesting that the addition of DD75 contributed to the dispersion of TiO_2_ nanoparticles.

**Figure 4:**
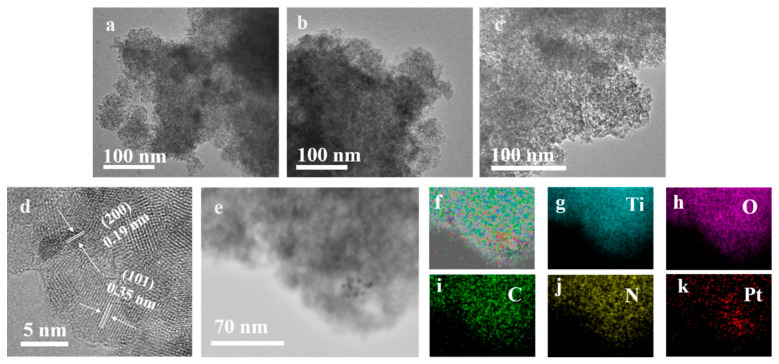
TEM images of (**a**) Pt-T200, (**b**) Pt-10%DD45-T200, and (**c**) Pt-10%DD75-T200; HRTEM image of (**d**) Pt-10%DD75-T200; (**e**–**k**) EDS elemental mapping images of Pt-10%DD75-T200.

Figure 4a–c present the TEM images of TiO_2_, Pt-10%DD45-T200, and Pt-10%DD75-T200, respectively. As can be seen, all of the samples consisted of nanoparticles. Moreover, the nanoparticles of Pt-10%DD75-T200 were more uniformly distributed than that of Pt-10%DD45-T200 and Pt-T200, which is in good agreement with the result of the SEM images. Clear diffraction patterns with interdistance d = 0.35 nm and d = 0.19 nm can be observed in the HRTEM image of Pt-10%DD75-T200 in Figure 4d, assigned to the (101) and (200) crystal planes of TiO_2_, respectively [34]. The average particle size values of Pt-T200, Pt-10%DD75-T200, and Pt-10%DD45-T200 nanoparticles were about 6 nm, indicating that the particle size of TiO_2_ was unchanged with the existence of chitosan and Pt. Furthermore, the EDS mapping analysis of Pt-10%DD75-T200 (Figure 4e–k) shows a coexistence and homogeneous dispersion of Ti, O, C, N, and Pt atoms in the nanoparticles, demonstrating the successful composition of TiO_2_ and chitosan, as well as the loading of Pt.

**Figure 5:**
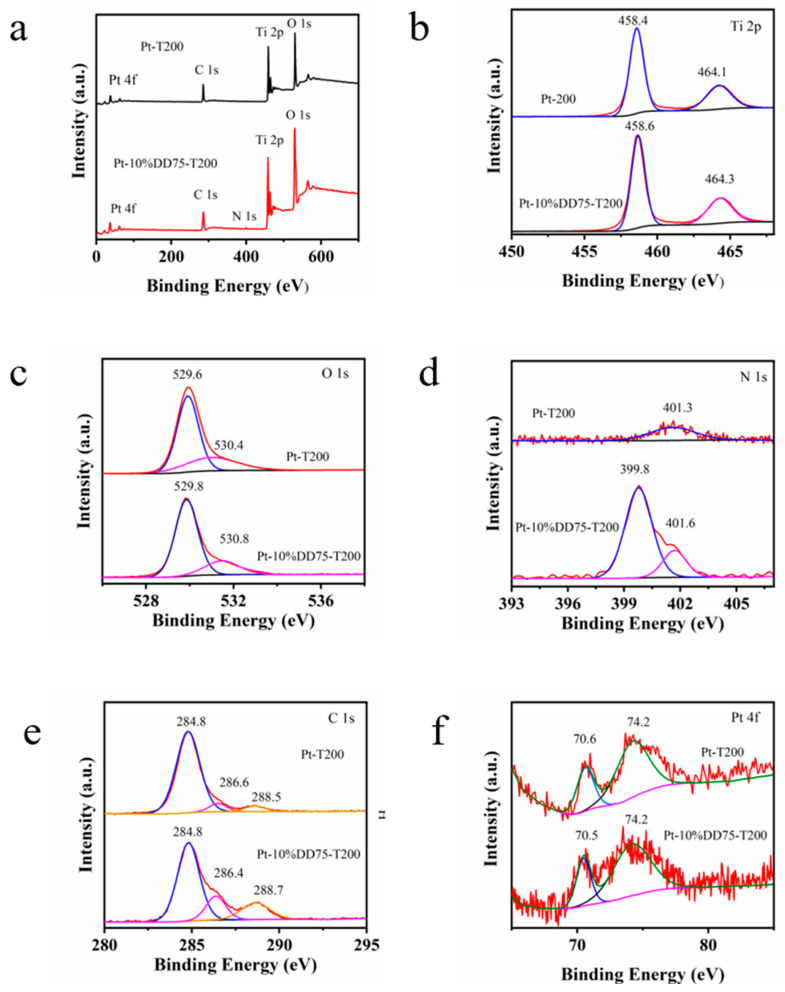
XPS spectra of Pt-10%DD75-T200: (**a**) survey, (**b**) Ti 2p, (**c**) O 1s, (**d**) N 1s, (**e**) C 1s, and (**f**) Pt 4f.

XPS analysis was employed to determine the specific bonding and chemical states of elements in Pt-T200 and Pt-10%DD75-T200 photocatalysts. As shown in Figure 5a, the wide-scan XPS spectrum of Pt-T200 illustrated the presence of Ti, O, C, and Pt elements; that of Pt-10%DD75-T200 illustrated the presence of Ti, O, N, C, and Pt elements. As shown in Figure 5b, the peaks located at 458.4 and 464.1 eV corresponded to Ti 2p_3/2_ and Ti 2p_1/2_, respectively, suggesting that the oxidation state of Ti was +4 [36]. As displayed in Figure 5c, the peaks located at 529.6 and 530.4 eV belonged to the lattice oxygen (Ti–O) and adsorbed oxygen (Ti–OH), respectively [8]. In Figure 5d, the N 1s peaks located at 399.8 eV were assigned to the N element contained in –NH_2_/–NH and –NH_3_^+^ groups in chitosan [36], while the peaks located at 401.3 eV originated from surface adsorbed or contaminated nitrogen species. The C 1s spectrum in Figure 5e displays the peaks located at 284.8, 286.4, and 288.7 eV, corresponding to the C signal in C–C/C=C bonds, C–OH and C=O, respectively [37]. The C1s signal for Pt-T200 was ascribed to the adsorbed carbon contaminants from the ambience, while the signal for Pt-10%DD75-T200 was due to the carbonaceous materials in chitosan. The Pt 4f high-resolution XPS spectrum shown in Figure 5f reveals two peaks at 70.5 and 74.2 eV, which were attributed to the Pt 4f_7/2_ and Pt 4f_5/2_ of Pt (0), respectively [38].

### 2.2. Photocatalytic H_2_ Evolution Performance

**Figure 6:**
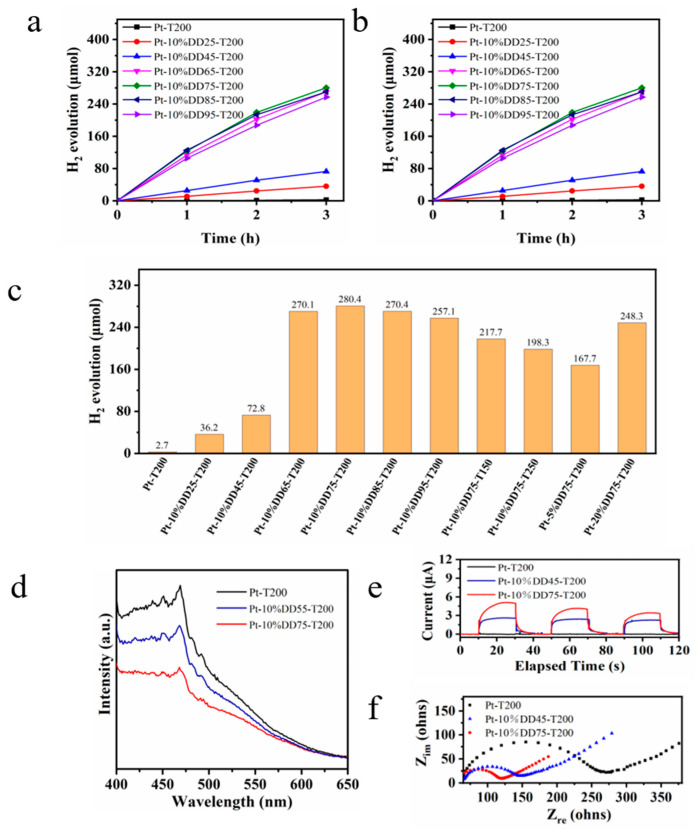
(**a**,**b**) Photocatalytic H_2_ evolution, (**c**) H_2_ evolution rates of as-prepared Pt-chitosan-TiO_2_ samples (λ > 420 nm, 10 vol% TEOA, pH = 11.3, 278 K), (**d**) emission spectra, (**e**) transient photocurrent (0.1M Na_2_SO_4_, Ag/AgCl electrode, Pt plate, sample electrodes, pH = 11.3, 0 V), (**f**) EIS Nyquist plots of Pt-T200, Pt-10%DD45-T200, and Pt-10%DD75-T200 (0.1M KCl, Ag/AgCl electrode, Pt plate, sample electrodes, pH = 11.3, 0.2 V).

The photocatalytic performance of the as-prepared samples was evaluated by water-splitting for H_2_ production under visible light, and the results are shown in Figure 6a–c. Very little H_2_ was detected over Pt-T200 (2.7 μmol) under visible-light irradiation as a result of its large band gap. A significant increase in photocatalytic activity was seen after the addition of chitosan. The evolution rates of H_2_ after 3 h of visible-light illumination for Pt-10%DDn-T200 (*n* = 25, 45, 65, 75, 85 and 95) were 36.2, 72.8, 270.1, 280.4, 270.4, and 257.1 μmol, respectively. The higher photocatalytic activity of Pt-10%DDn-T200 (*n* = 65, 75, 85 and 95) compared with that of Pt-10%DDn-T200 (*n* = 25 and 45) may have been due to the solubility of DDn in titanium sol being improved with the increase in the degree of deacetylation. This facilitated the condensation of the hydroxyl groups in both chitosan and TiO_2_ and aided the formation of the CT complex in the process of calcination, leading to the outstanding visible-light activity. Furthermore, the formation of CT complexes between TiO_2_ and chitosan could absorb visible-light through the LMCT mechanism, leading to the outstanding visible-light activity. When the deacetylation degree of chitosan reached 75%, Pt-10%DD75-T200 showed an optimal photocatalytic activity for water reduction. The effects of calcination temperature and the amount of chitosan on the photocatalytic activity of Pt-mDD75-Tt (m = 5%, 10%, and 20%) were investigated. After 3 h of visible-light illumination, the evolution rates of H_2_ for Pt-10%DD75-Tt (*t* = 150, 200 and 250 °C) were 217.7, 280.4, and 198.3 μmol, respectively. The lower photocatalytic activity of the sample calcinated at 150 °C could have been due to an incomplete conversion from titanium hydroxide to TiO_2_ and a lower crystallinity of TiO_2_. A calcination temperature of 250 °C led to the partial decomposition of chitosan and, consequently, the weak photocatalytic activity. The performance of Pt-10%DD75-T200 was also much higher than that of Pt-5%DD75-T200 (167.7 μmol^1^) and Pt-15%DD75-T200 (248.3 μmol). The photocatalytic activity improved with increasing amounts of chitosan due to increasing amounts of CT complexes. When the amount of chitosan was further increased, the photocatalytic activity decreased. This may have been due to excess chitosan occupying the photocatalytically active sites of TiO_2_. Control experiments for photocatalytic H_2_ evolution reaction over Pt-10%DD75-T200 are shown in Figure S2. The figure demonstrates that no H_2_ could be detected in the absence of TEOA, irradiation and a photocatalyst. Figure S3 presents the action spectra of Pt-10%DD75-T200. As can be seen, the apparent quantum efficiencies at the different illumination wavelengths of Pt-10%DD75-T200 were in good accordance with the absorbance spectrum. Furthermore, the FT-IR and XPS spectra of Pt-10%DD75-T200 before and after irradiation are shown in Figures S4 and S5, which indicated that the photocatalyst barely changed and confirmed its stability.

Photoluminescence spectroscopy was applied to study the migration, transfer, and recombination of electron–hole pairs generated by the photocatalyst. The excitation spectra for Pt-T200, Pt-10%DD45-T200, and Pt-10%DD75-T200 is shown in Figure S6 with the emission wavelength at 350 nm, which exhibited excitation peaks appearing at 358 nm. As presented in Figure 6d, the emission intensity followed the order of Pt-10%DD75-T200 < Pt-10%DD45-T200 < Pt-T200, which was opposite to the order of photocatalytic activity. The lower PL intensity was related to the slower recombination rate of electron-hole pairs. The photocurrent response curves and EIS Nyquist plots of Pt-T200, Pt-10%DD45-T200, and Pt-10%DD75-T200 are shown in Figure 6e,f. As can be seen, Pt-10%DD75-T200 showed the strongest photocurrent density and smallest semicircle, indicating the highest photo-induced charge transfer and separation efficiency. In conclusion, these commendable photo- and electro-chemical characterizations support that the combination of chitosan and TiO_2_ leads to efficient interfacial charge transfer and the suppression of photoexcited charge recombination.

### 2.3. Mechanism of Photocatalytic Reaction

**Figure 7:**
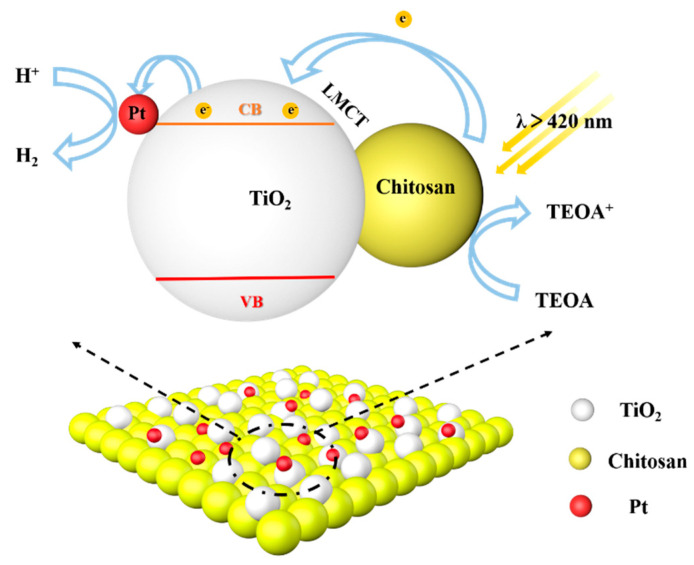
A schematic illustration of the photocatalytic mechanism for H_2_ production through water reduction over a Pt-chitosan-TiO_2_ charge transfer complex.

Based on the above analysis and experimental results, a possible photocatalytic mechanism for water reduction over Pt-chitosan-TiO_2_ CT complex photocatalyst was proposed (Figure 7). In this process, chitosan attaches to the TiO_2_ surface through their hydroxyl groups, sensitizing the TiO_2_ by forming LMCT complexes. Typically, electrons can be easily excited from the HOMO level of chitosan and transferred to the CB of TiO_2_ under visible-light irradiation. In addition, due to a higher work function of Pt (5.65 eV), electrons are transferred to the Pt cocatalyst and then react with H^+^ to form H_2_. TEOA plays a role as a hole scavenger for the reaction.

## 3. Materials and Methods

### 3.1. Materials

Chitin, N,N-dimethylformamide, hydrochloric acid (HCl), and chloroplatinic acid hexahydrate (H_2_PtCl_6_·6H_2_O) were purchased from Aladdin (Shanghai, China). Methyl orange (MO), sodium hydroxide (NaOH), and sodium borohydride (NaBH_4_) were provided by Sinopharm (Shanghai, China). Triethanolamine (TEOA) was purchased from Tianjin Fuchen Chemical Reagent Factory (Tianjin, China). Titanium tetraisopropoxide (C_12_H_28_O_4_Ti) was provided by Sigma-Aldrich (Shanghai, China). All solutions were prepared using deionized (DI) water.

### 3.2. Preparation of Photocatalysts

Chitosan with different degrees of deacetylation was prepared by a concentrated alkali method [39]. We uniformly dispersed 1 g chitin in 25 mL of 50% NaOH solution. After that, the solution was stirred at 90 °C for a fixed length of time. The reacted mixtures were washed with deionized water for eliminating the excess lye until the pH of the solution was neutral. The obtained products were then placed in an oven at 80 °C and dried for 8 h. Finally, different deacetylation degrees of chitosan samples were obtained and denoted as DDn, where *n* reflects the degree of deacetylation of chitosan.

Chitosan-TiO_2_ complexes were synthesized by a sol-gel method. Titanium sol was formed by hydrolyzing titanium tetraisopropoxy under acidic conditions and dialyzing the suspension to pH~4 [40]. Calcination of 100 mL of TiO_2_ sol yielded 3 g of powder. To obtain mDDn-TiO_2_ (where m = 5%, 10%, or 20%), DDn (0.0355, 0.075, and 0.169 g) was dispersed in 25 mL of titanium sol and 10 mL of deionized water and then mechanically stirred at ambient temperature. After being dried in a microwave, the gels were calcined at different temperatures (150, 200, or 250 °C) in a muffle furnace and maintained for 3 h. Finally, samples of mDDn-Tt were obtained, where m is the content of chitosan, T is the TiO_2_, and t is the calcination temperature.

Pt-mDDn-Tt samples were synthesized by an impregnation method. We dispersed 1 g of DDn-TiO_2_ in 1.34 mL of chloroplatinic acid hexahydrate (H_2_PtCl_6_·6H_2_O; 10 mg·mL^−1^) by sonication for 30 s. Then, 2 mL of mixed aqueous solution containing 0.1 mol·mL^−1^ of sodium borohydride (NaBH_4_) and 0.1 mol·mL^−1^ of sodium hydroxide (NaOH) was added. After that, the mixture was sonicated for 30 s. The mixture was then filtered, washed, dried, and ground to obtain the Pt-mDDn-Tt photocatalyst.

### 3.3. Determination of Degree of Deacetylation

The degree of deacetylation of chitosan was evaluated via an acid-base titration. Chitosan (0.25 g) was dissolved in 20 mL 0.1 M of HCl at 25 °C, and then two drops of MO indicator were added. 0.1 M of NaOH was used to titrate the solution until the color of the solution changed to orange-yellow. The degree of deacetylation of chitosan was calculated using:

NH_2_% = [(C_1_V_1_ − C_2_V_2_) × 0.016]/[G (100 − W)] × 100

DD% = NH_2_%/9.94% × 100%

where C_1_ is the concentration of HCl (M); C_2_ is the concentration of NaOH (M); V_1_ is the volume of HCl added (mL); V_2_ is the volume of NaOH added by titration (mL); G is the sample weight (g); and W is the sample water content (%), 0.016, equal to NH_2_ content (g) in 1 mL of 1 M HCl, 9.94%: chitosan theoretical NH_2_ content.

### 3.4. Characterization

Thermogravimetric analysis (TGA) was recorded on a Netzsch Sta 449C (Netzsch, Selb, Germany) thermal analysis instrument. The X-ray diffraction (XRD) patterns of samples were performed on a Bruker D8 Advance X-ray diffractometer (D8 Advanve, Bruker, Ettlingen, Germany) with Cu-Kα radiation, running at an accelerating voltage of 40 kV and applied current of 40 mA. BaSO_4_ was employed as a reference to record UV–visible diffuse reflectance spectra (DRS) on a Varian Cary 500 apparatus (Varian, Palo Alto, CA, USA) equipped with an integrating sphere. The FT-IR spectra of samples were studied using a Nicolet Nexus 670 FT-IR spectrometer (ThermoScientific, Waltham, MA, USA). BELSORP-mini II equipment (MicrotracBEL, Osaka, Japan) was used to study the N_2_ adsorption-desorption isotherms and BJH pore size distribution of samples. The morphology of the as-prepared samples was captured by field-emission scanning electron microscopy (FESEM, JEOL JSM-7500F, JEOL, Tokyo, Japan), operated at 5 kV and 10 μA. TEM, high-resolution transmission electron microscopy (HRTEM), and elemental mapping images were filmed by a JEOL model JEM 2100 F instrument (JEOL, Tokyo, Japan) at an accelerating voltage of 200 kV. A monochromatic Al-Kα source was employed to perform the X-ray photoelectron spectroscopy (XPS) of the samples on a PHI Quantum 2000 system. Photoluminescence (PL) measurements were analyzed on a FLSP920 (EI) fluorescence spectrophotometer (Edinburgh Instruments, Livingston, UK). A 20 mg sample was used for tableting, and the excitation spectra were measured with an emission wavelength at 350 nm.

### 3.5. Photocatalytic Measurements

The photocatalytic H_2_ evolution was carried out in a glass-closed photocatalytic activity evaluation system (Labsolar-6A, Perfectlight). A 300 W Xe lamp (PLS-SXE300/300UV, Perfectlight, Beijing, China) was used as the light source (λ ≥ 420 nm). We dispersed 100 mg of as-synthesized sample in 100 mL of 10 vol% aqueous triethanolamine (TEOA) solution in a quartz reactor at a constant temperature of 5 °C. Before the irradiation, the evaluation system was degassed. The amount of H_2_ produced from the water reduction was analyzed by a gas chromatograph (GC 9790, FuLi, China) equipped with a TCD detector.

### 3.6. Photoelectrochemical Measurements

The powder sample was prepared on fluorine-doped tin oxide (FTO) glass. We sonicated 5 mg of the sample in 0.5 mL of N,N-dimethylformamide to disperse it evenly. After that, the paste was spread onto the conductive surface of the FTO glass and then dried. All the photoelectrochemical measurements were tested in a three-electrode cell with a Pt plate and Ag/AgCl electrodes as the counter and reference electrodes, and the sample electrode as the working electrode. The electrochemical impedance spectroscopy (EIS) measurement and transient photocurrent were performed using an electrochemical workstation (Versa STAT3, Princeton Instruments, Acton, MA, USA) with 0.1 M KCl and 0.1 M Na_2_SO_4_ as the electrolyte. A 300 W Xe lamp (PLS-SXE300/300UV, Perfectlight) and UV cutoff filter (λ > 420 nm) were used as the radiation source.

## 4. Conclusions

In summary, a new Pt-chitosan-TiO_2_ CT complex was synthesized via a sol-gel and impregnation method. The optical absorbance range of TiO_2_ was expanded to the visible region, attributed to the LMCT mechanism that allows photo-excited electrons from the HOMO of chitosan to transfer to the conduction band of the TiO_2_. By optimizing the preparation conditions of the photocatalysts (the degree of deacetylation of chitosan, additional amounts of chitosan and calcination temperature), the synthesized Pt-10%DD75-T200 showed outstanding photocatalytic activity (280.4 μmol) for H_2_ evolution from water reduction after 3 h of visible-light irradiation. The underlying reaction mechanism for water reduction over a Pt-chitosan-TiO_2_ CT complex under visible-light irradiation was proposed. This work provides a new idea for converting natural polysaccharide biomass waste into attractive functional materials, showing potential applications in visible-light-driven H_2_ evolution from water reduction.

## Data Availability

The data presented in this study are available upon request from the corresponding author.

## Supplementary Materials

The following supporting information can be downloaded at: https://www.mdpi.com/article/10.3390/molecules27154673/s1, Figure S1: UV-vis diffuse reflectance spectra of as-prepare Pt-Chitosan-TiO_2_ samples; Figure S2: Control experiments for photocatalytic H_2_ evolution reaction over Pt-10%DD75-T200; Figure S3: Action spectra of Pt-10%DD75-T200 (illumination time: 1 h, 10 vol% TEOA, pH = 11.3, 278 K); Figure S4: FT-IR spectra of Pt-10%DD75-T200 before and after irradiation; Figure S5: XPS spectra of Pt-10%DD75-T200 before and after irradiation; Figure S6: Excitation spectra of Pt-T200, Pt-10%DD45-T200 and Pt-10%DD75-T200; Table S1: UV-vis diffuse reflectance spectra of (a) DD45, DD75 and (c) as-prepare Pt-Chitosan-TiO_2_ samples.

## Sample Availability

Samples of the compounds are not available from the authors.
