# Supplementary material for: Pt-Chitosan-TiO2 for Efficient Photocatalytic Hydrogen Evolution via Ligand-to-Metal Charge Transfer Mechanism under Visible Light
Source: Molecules. 2022 Jul 22;27(15):4673. doi: 10.3390/molecules27154673 (PMC9330878; doi:10.3390/molecules27154673)
Supplement: Supplementary file 1 [file molecules-27-04673-s001.zip › molecules-1758141-supplementary.pdf]

Table S1. Table of the samples (pt-mDDn-Tt)

Sapmle ID	titanium sol (mL)	Chitosan (g)	degree of deacetylation (%)	Temperature (°C)	Time (h)
1# Pt-T200	25	0	/	200	3
2# Pt-10%DD25-T200	25	0.075	25	200	3
3# Pt-10%DD45-T200	25	0.075	45	200	3
4# Pt-10%DD65-T200	25	0.075	65	200	3
5# Pt-10%DD75-T200	25	0.075	75	200	3
6# Pt-10%DD85-T200	25	0.075	85	200	3
7# Pt-10%DD95-T200	25	0.075	95	200	3
8# Pt-5%DD75-T200	25	0.0355	75	200	3
9# Pt-20%DD75-T200	25	0.169	75	200	3
10# Pt-10%DD75-T150	25	0.075	75	150	3
11# Pt-10%DD75-T250	25	0.075	75	250	3

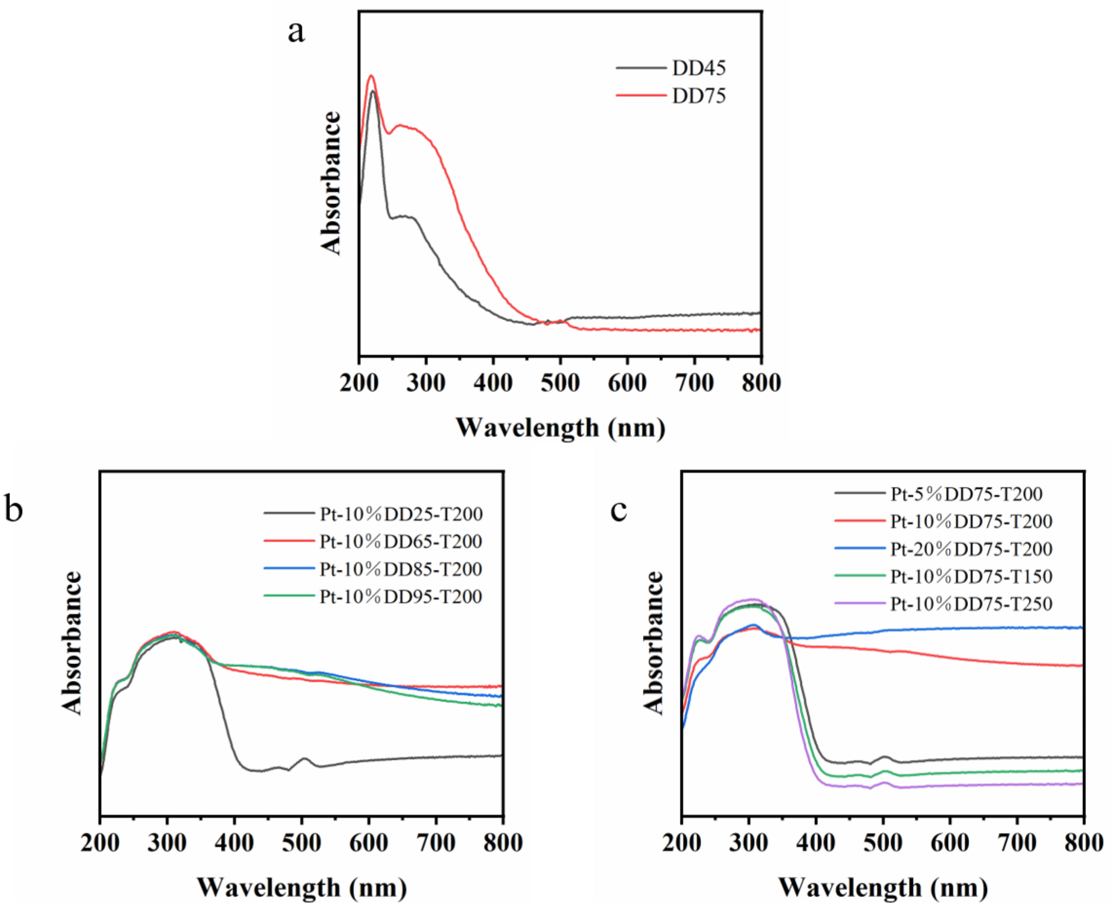

Figure S1. UV-vis diffuse reflectance spectra of as-prepare Pt-Chitosan-TiO_2 samples.

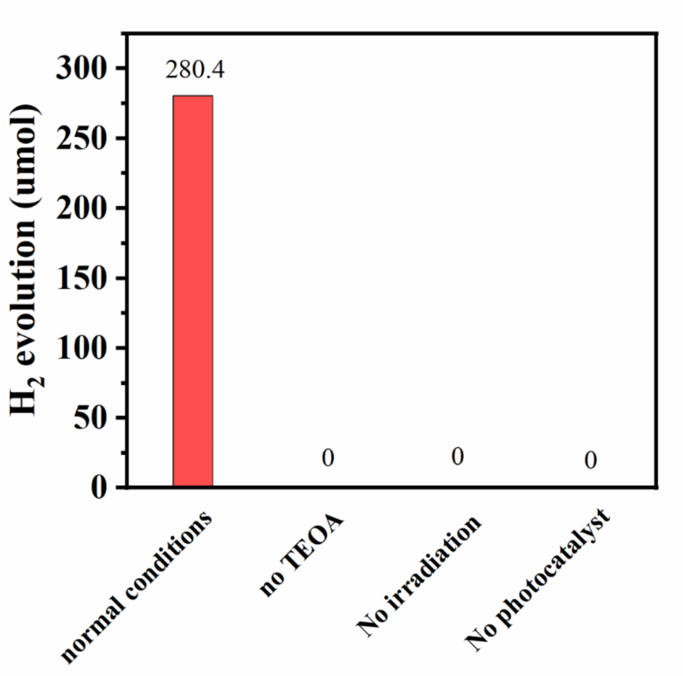

Figure S2. Control experiments for photocatalytic H_2 evolution reaction over Pt-10%DD75-T200.

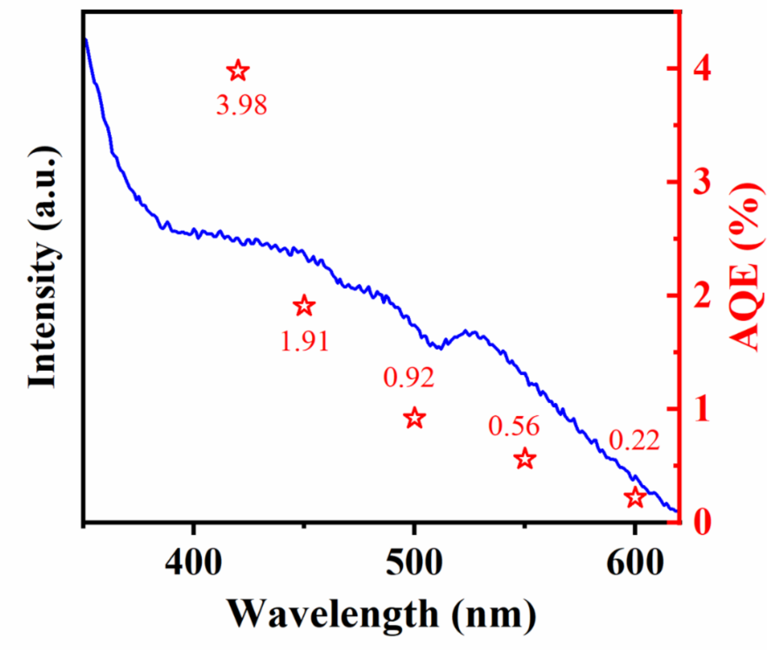

Figure S3. Action spectra of Pt-10%DD75-T200 (illumination time: 1h, 10 vol% TEOA, pH = 11.3, 278 K).

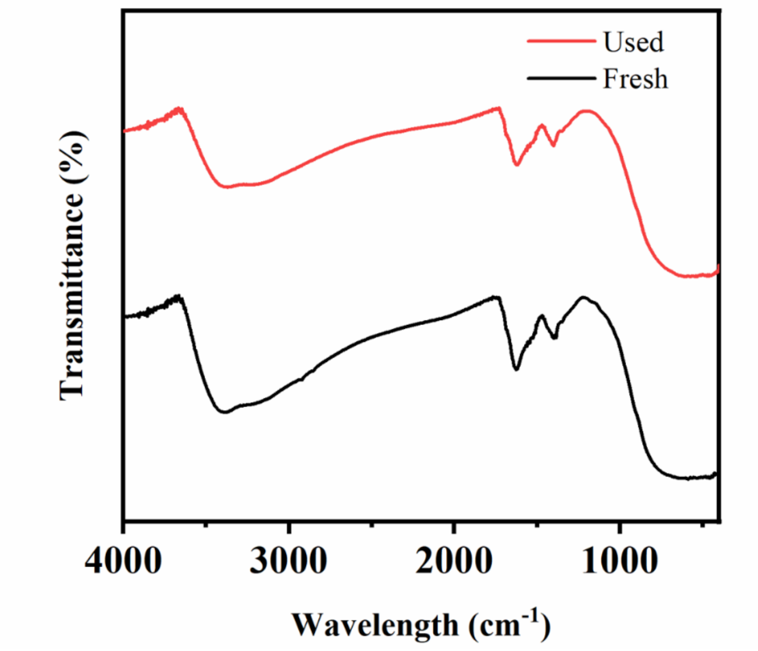

Figure S4. FT-IR spectra of Pt-10%DD75-T200 before and after irradiation.

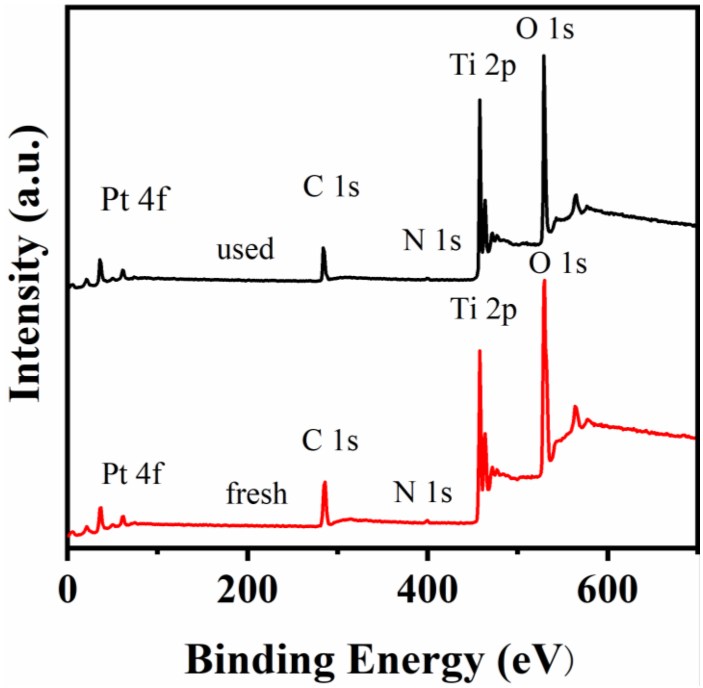

Figure S5. XPS spectra of Pt-10%DD75-T200 before and after irradiation.

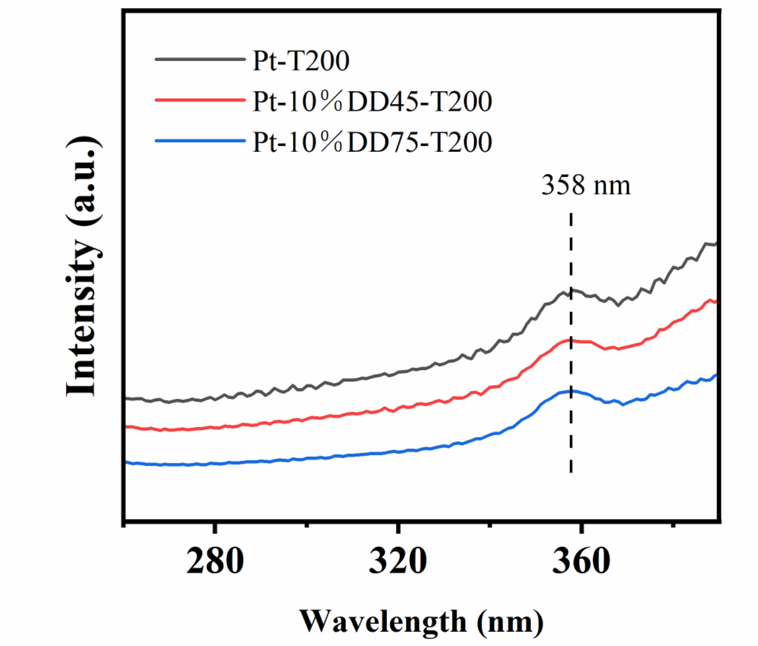

Figure S6. Excitation spectra of Pt-T200, Pt-10%DD45-T200 and Pt-10%DD75-T200.
